# An Anti-Influenza Virus Antibody Inhibits Viral Infection by Reducing Nucleus Entry of Influenza Nucleoprotein

**DOI:** 10.1371/journal.pone.0141312

**Published:** 2015-10-29

**Authors:** Aerin Yoon, Kye Sook Yi, So Young Chang, Sung Hwan Kim, Manki Song, Jung Ah Choi, Melissa Bourgeois, M. Jaber Hossain, Li-Mei Chen, Ruben O. Donis, Hyori Kim, Yujean Lee, Do Been Hwang, Ji-Young Min, Shin Jae Chang, Junho Chung

**Affiliations:** 1 Department of Biochemistry and Molecular Biology, Seoul National University College of Medicine, Seoul National University, Seoul, South Korea; 2 Cancer Research Institute, Seoul National University College of Medicine, Seoul National University, Seoul, South Korea; 3 Biotechnology Research Institute, Celltrion Inc., Incheon, South Korea; 4 Institut Pasteur Korea, Gyeonggi-do, South Korea; 5 International Vaccine Institute, Seoul, South Korea; 6 Influenza Division, Centers for Disease Control and Prevention, Atlanta, GA, United States of America; Lindsley F. Kimball Research Institute, UNITED STATES

## Abstract

To date, four main mechanisms mediating inhibition of influenza infection by anti-hemagglutinin antibodies have been reported. Anti-globular-head-domain antibodies block either influenza virus receptor binding to the host cell or progeny virion release from the host cell. Anti-stem region antibodies hinder the membrane fusion process or induce antibody-dependent cytotoxicity to infected cells. In this study we identified a human monoclonal IgG_1_ antibody (CT302), which does not inhibit both the receptor binding and the membrane fusion process but efficiently reduced the nucleus entry of viral nucleoprotein suggesting a novel inhibition mechanism of viral infection by antibody. This antibody binds to the subtype-H3 hemagglutinin globular head domain of group-2 influenza viruses circulating throughout the population between 1997 and 2007.

## Introduction

Influenza virus is an enveloped RNA virus with two major surface integral-membrane glycoproteins, hemagglutinin (HA) and neuraminidase (NA). Membrane-envelope HA consists of two disulfide-linked glycosylated polypeptides, HA1 and HA2 [1]. The major part of HA1 forms the globular head domain and binds to sialic acid receptors on the host cell plasma membrane; whereas, HA2 forms most of the HA stem region and induces pH-triggered membrane fusion between the influenza-virus envelope and host-cell endosomal membranes [2]. The NA protein is crucial for destroying sialic acid-containing receptors on the host cell and viral membranes, permitting progeny virion release from infected cells [3]. Currently, 17 HA and 10 NA subtypes have been identified, and strains of influenza A virus are classified by subtype according to their surface glycoproteins [1, 2]. Three HA subtypes (H1, H2, and H3) and two NA subtypes (N1 and N2) have caused extensive influenza outbreaks in humans [4]; in particular, H1N1 and H3N2 influenza viruses are the main causes of seasonal influenza outbreaks [5].

Neutralizing antibodies play a critical role in protecting the host cell from influenza virus infection. The presence of host-cell antibodies against either HA or NA reduces influenza virus infectivity [6–11]. The HA globular head domain is the major antigenic component on the influenza virus surface. Anti-HA antibodies can neutralize the influenza virus by preventing either of HA’s two functions, i.e., mediating influenza virus attachment to, and membrane fusion with, the host cell [12]. The presence of anti-HA globular-head-domain antibodies drives the outgrowth of antigenic variants, resulting in a continuum of changes in HA structure in viral progeny, known as antigenic drift [13]. Diversity of HA sequences of influenza A virus is high. There are 17 HA serotypes belonging to one of two major categories: group 1 (H1, H2, H5, H6, H8, H9, H11, H12, H13, H16, and H17) or group 2 (H3, H4, H7, H10, H14, and H15) [14].

A minor portion of anti-HA antibodies target the HA stem region, and some of these antibodies can neutralize the influenza virus by inhibiting membrane fusion [13, 15]. Because the stem region is highly conserved among influenza viruses, antibodies reacting with the HA stem region tend to be broadly neutralizing against viral infectivity [16].

In this study, we constructed a phage-display combinational antibody library using B cells obtained from influenza-vaccinated volunteers. From this library, we selected neutralizing anti-H3 antibodies, one of which neutralized only H3N2 viruses collected between 1997 and 2007, indicating its binding is vulnerable to antigenic drift. Altering seven residues in the H3N2 HA globular head domain of strains isolated in 1997 to match sequences of strains isolated in 1995 abolished our selected antibody’s reactivity. These observations suggested that the binding site of this antibody is localized in the HA globular head domain. Interestingly, this antibody inhibits neither the receptor binding nor the membrane fusion process. But the antibody efficiently reduced the nucleus entry of viral nucleoprotein. To the limit of our knowledge, this is the first report on an antibody with a novel inhibitory mechanism of influenza virus infection not reported hitherto.

## Materials and Methods

### Ethics Statement

The mouse studies conducted at CDC were performed in accordance to protocols approved by the Institutional Biosafety Committee and Animal Care and Use Committee (Protocol #2069). Mice were anesthetized by inhalation of 5% isoflurane/95% O_2_ before the virus infection to minimize suffering and monitored daily for clinical signs and body weight recordings (S1 ARRIVE Checklist). Animals that exhibited mild or moderate clinical signs were observed twice per day, whereas animals that exhibited severe clinical illness were humanely euthanized. Animals whose body weight reached 75% or less of their initial weight were humanely euthanized immediately. All mice that reached the pre-established euthanasia endpoint were euthanized by an overdose of anesthetic (isoflurane).

All ferret research procedures were reviewed and approved by the IACUC of Bioleaders (#BLS-ABSL-10-021). Ferrets were monitored daily for clinical signs of infection. Furthermore, all infections as well as sample collections including blood and nasal rinsed solution were performed under zoletil/xylazine cocktail anesthesia, minimizing animal suffering. For the collection of lung tissue, ferrets were humanely euthanized by an overdose of anesthetic (zoletil/xylazine).

### Library construction and biopanning

Total RNA was prepared using TRI Reagent® (Molecular Research Center, Inc., Cincinnati, OH, USA) from the peripheral blood mononuclear cells of 13 volunteers who had been vaccinated against the A/Uruguay/716/2007 H3N2 strain. All subjects provided the written informed consent to participate in this study. The protocol was approved by the institutional Review Board, Gangnam Severance Hospital Yonsei University College of Medicine, Seoul, Korea (Permit #4-2009-0683) and the study was carried out in strict accordance with the ethical guidelines of Gangnam severance hospital.

First-strand cDNA was synthesized using SuperScript^TM^ reverse transcriptase with oligo (dT) priming (Invitrogen, Grand Island, NY, USA). Using this cDNA, a phage-display library of human single-chain variable fragments (scFv) was constructed using the pComb3XSS phagemid vector as previously described [17]. Four rounds of panning were performed to select scFv clones from the library [17]. For each round of biopanning, 1.5 μg recombinant His-tagged trimeric HA protein from the A/Brisbane/10/2007 strain (Influenza Reagent Resource, Manassas, VA, USA) was used to coat 5×10^6^ magnetic beads (Dynabeads M-270 epoxy) (Invitrogen) used for scFv retrieval.

### Enzyme immunoassay

Production and purification of CT302 IgG_1_ were performed as described previously [18]. Recombinant His-tagged trimeric HA protein from the A/Brisbane/10/2007 strain (5, 10, 50, 100, or 200 ng) dissolved in 20 μl phosphate-buffered saline (PBS) was added to microtiter plate wells and incubated at 4°C overnight. Plates were washed three times with PBST (PBS containing 0.05% v/v Tween 20) and incubated for 1 hr at 37°C with 3% (w/v) bovine serum albumin (BSA) in PBS to block nonspecific antibody binding. After washing plates with PBST, 50 ng CT302 IgG_1_ or control human anti-respiratory syncytial virus IgG_1_ (palivizumab) (MedImmune Inc., Gaithersburg, MD, USA) was dissolved in 50 μl 3% (w/v) BSA in PBS and added to each well. After incubation for 1 hr at 37°C, followed by two washes with PBST, plates were incubated with a commercially available horseradish peroxidase (HRP)-conjugated anti-human IgG_1_ solution (Thermo Fisher Scientific, Waltham, MA, USA) diluted 5,000-fold in 3% BSA in PBS. After washing with PBST, 50 μl HRP colorimetric substrate solution (ABTS) (Amresco Inc., Solon, OH, USA) was added to each well, and optical density was measured at 405 nm with a microtiter-plate reader (Labsystems S.L., Barcelona, Spain).

### Immunoblot analysis

One microgram recombinant His-tagged trimeric HA proteins from H1, H3, and H5 strains (Influenza Reagent Resource) were dissolved and boiled in Laemmli sample buffer without 2-mercaptoethanol, electrophoresed on 4–12% (w/v) Tris-glycine gradient gels (Novex® NuPAGE®) (Invitrogen), and transferred to two different nitrocellulose membranes. Membranes were blocked with Tris-buffered saline containing Tween 20 (TTBS: 10 mM Tris/HCl, pH 7.5; 150 mM NaCl; and 0.05% v/v Tween 20) and 5% (w/v) skim milk (TTBS-M) at room temperature for 30 min. One membrane was probed with 10 μg/ml CT302 IgG_1_ in TTBS-M at room temperature for 2 hr. The membrane was washed with TTBS and incubated at room temperature for 1 hr with HRP-conjugated anti-human IgG (Thermo Fisher Scientific) diluted 5,000-fold in TTBS-M. The other membrane was blocked and incubated at room temperature for 1 hr with HRP-conjugated rabbit anti-His IgG (Pierce, Rockford, IL, USA) diluted 5,000-fold in TTBS-M. Both membranes were washed three times with TTBS, and protein bands were visualized using SuperSignal® West Pico Chemiluminescent Substrate (Pierce) following the manufacturer’s instructions.

### Sequence analysis

Phagemid DNA of selected clones identified by enzyme immunoassays was prepared with a small-scale plasmid preparation kit (Qiagen, Hilden, Germany). Sequence analysis of positive clones was performed as previously described [17]. Briefly, the OmpSeq primer (5'-AAGACAGCTATCGCGATTGCAG-3') was used to sequence the V_H_ and V_L_ chains of the CT302 IgG_1_ [17], and Accelrys Gene software (Accelrys Inc.; San Diego, CA, USA) was used to search for homologs of selected clones.

### Real-time interaction analysis

Kinetic interactions between CT302 IgG_1_ and recombinant His-tagged trimeric HA protein of A/Brisbane/10/07 or A/Wisconsin/67/2005 strains were determined using the BIAcore™T200 system (GE Healthcare, Uppsala, Sweden) [19]. First, anti-His antibody (Ab Chem, Montreal, Canada) was dissolved (6 μg/ml) in 10 mM sodium acetate buffer (pH 4.5) and immobilized on a sensor chip, consisting of a gold surface with covalently attached carboxymethylated dextran (CM5 dextran sensor chip) (GE Healthcare), at a flow rate of 5 μl/min using the Amine Coupling Kit (GE Healthcare). Then recombinant His-tagged trimeric HA protein was dissolved at a final concentration of 12 μg/ml in HEPES-buffered saline containing 0.005% surfactant P20, 3 mM EDTA, and 0.15 M NaCl. Subsequently, recombinant His-tagged trimeric HA protein was injected for 30 sec at a flow rate of 10 μl/min in order to capture recombinant His-tagged trimeric HA protein with anti-His antibody. The CT302 IgG_1_ was dissolved in HEPES-buffered saline, serially diluted 3-fold over the range from 100 to 1.23 nM, and injected over 150 sec into the BIAcore system to bind the immobilized recombinant His-tagged trimeric HA protein on the sensor chip, at a flow rate of 30 μl/min at 25°C. Dissociation of CT302 IgG_1_ from the immobilized recombinant His-tagged trimeric HA protein was monitored after the end of the association phase at a flow-rate of 30 μl/min. The sensor chip surface was regenerated with 10 mM glycine (pH 2.0). Evaluation software for BIAcore T-200 version 1.0 (GE Healthcare) was used to calculate *K*_on_ and *K*_off_ constants. The BIAcore system was also used to confirm if there was any competition between CT302 IgG_1_ and CT149 IgG_1_ (an anti-HA stem-region IgG_1_, unpublished results) in binding to HA protein from the A/Brisbane/10/2007 strain. By immobilizing anti-His antibody on a sensor chip, recombinant His-tagged trimeric HA protein from A/Brisbane/10/2007 strain was allowed to interact with the anti-His antibody. Next, either CT302 IgG_1_ or CT149 IgG_1_ (diluted to 100 nM) was injected across the surface to observe the interaction between recombinant His-tagged trimeric HA protein from A/Brisbane/10/2007 strain and CT302 IgG_1_ or CT149 IgG_1_. The amount of test material bound to the sensor chip surface in the real-time interaction experiments was expressed in arbitrary resonance units (RU). One RU represents approximately 1 pg protein/mm^2^ of the sensor chip surface [20].

### Microneutralization assay

The microneutralization assay was performed as previously reported using purified CT302 IgG_1_ with a minor modification [21–23]. Briefly, this assay tests an antibody’s ability to inhibit influenza virus infection of Madin-Darby canine kidney (MDCK-L) cells *in vitro* [24]. Influenza viral strains A/Hong Kong/1968, A/Beijing/353/1989-X109, A/Beijing/32/1992-R, A/Johannesburg/33/1994-R, A/Nanchang/933/1995, A/Sydney/5/1997, A/Panama/2007/1999, A/Wyoming/3/2003.rg, and A/Brisbane/10/2007 were employed for the assay. Handling of influenza virus was performed in a biosafety level-2 containment laboratory.

Two-fold serial dilutions of CT302 IgG_1_ in Dulbecco’s Modified Eagle Medium (DMEM) supplemented with 0.3% w/v BSA (Life Technologies), penicillin and streptomycin, and 25 mM HEPES were mixed with 100 TCID_50_ of influenza virus in 96-well Costar plates and incubated for 1 hr at 37°C. During this incubation, plates were shaken at 20-min intervals to ensure resuspension. The TCID_50_ value denotes the tissue-culture infective dose required to cause cytopathic effects in 50% of inoculated cells. MDCK-L cells (3×10^4^/well) were then added in the presence of 1 μg/ml trypsin (TPCK-trypsin) (Sigma-Aldrich; St. Louis, MO, USA), and plates were incubated at 37°C under 5% CO_2_ for 20 hr. After incubation, culture supernatants were removed, cells were fixed with 80% (v/v) cold acetone in PBS, and plates were processed for detection of viral proteins with a cell-based ELISA system. A biotin-labeled monoclonal antibody specific for influenza A virus (Millipore Corp.; Billerica, MA, USA) was added to each well (100 μl of a 1:2,000 dilution in 1% w/v BSA containing PBST) and incubated for 1 hr at room temperature. Plates were washed with PBST, and streptavidin-HRP conjugate (Millipore) was added (100 μl of a 1:15,000 dilution in 1% w/v BSA containing PBST) to each well. After 1 hr incubation at room temperature, plates were washed and developed with 100 μl O-phenylenediamine chromogenic substrate for 10 min. The reaction was stopped with 50 μl of 3N HCl, and the absorbance was measured at 490 nm using a microtiter-plate reader (Labsystems S.L.). The microneutralization titer was determined according to previously described methods [25].

### Hemagglutination inhibition assay

The hemagglutination inhibition assay was performed as previously described [26]. Briefly, 20 μg/ml purified CT302 IgG_1_ was serially diluted 2-fold in PBS and combined with 8 hemagglutination units of influenza virus in 96-well cell culture plates. Four strains of influenza virus were tested: A/Brisbane/10/2007, A/Sydney/5/1997, A/Philippines/2/1982, and A/Hong Kong/1968. The influenza viruses were propagated in 10-day-old embryonated chicken eggs. After a 1-hr preincubation of antibody with influenza virus, chicken red blood cells (RBCs) were added to a final concentration of 0.5%, the plate was incubated for 1 hr at room temperature, and hemagglutination was evaluated by light microscopy.

### Cell fusion assay

The cell fusion assay was performed as previously described [13]. Briefly, Chinese hamster ovary (CHO) cells (Invitrogen) that overexpress HA protein from A/Brisbane/10/2007 strain were plated at approximately 90% confluence in six-well plates. After incubation at 37°C for 24 hr in DMEM with 10% (v/v) fetal bovine serum (FBS), cells were washed with serum-free DMEM and incubated for 30 min. Cells were washed with serum-free DMEM again and incubated with 5 μg/ml tolylsulfonyl phenylalanyl chloromethyl ketone (TPCK)-trypsin in serum-free DMEM for 5 min. Subsequently, trypsin was neutralized by adding FBS to a final concentration of 10%. Cells were incubated for 30 min with 10 μg/ml CT302 IgG_1_, washed with PBS, and incubated with low-pH fusion-inducing buffer (150 mM NaCl buffered to pH 5.0 with 10 mM HEPES). Cells were returned to DMEM with 10% FBS and incubated 2–3 hr at 37°C. Finally, cells were fixed with ice-cold methanol and stained with trypan blue.

### Viral entry assay

Entry fusion (EF) and nuclear import (EI) assay were carried out as previously described [27, 28]. In the EF assay, membrane fusion between the virus envelope and endosomal membrane was observed using A/Brisbane/10/2007 virus labeled with the lipophilic fluorescent dyes R18 and SP-DiOC18 (Molecular Probes/ Thermo Fisher Scientific, MA, USA). Then labeled viruses were pre-incubated with 12.5 mg/ml CT302 IgG_1_ or anti-respiratory syncytial virus IgG_1_ (palivizumab) (MedImmune Inc.) at 37 °C for 30 min. A549 cells grown to 50% confluence in 96-well μ clear-plate, black were inoculated with R18/DiOC18-labeled and antibody pre-incubated A/Brisbane/10/2007 virus (MOI of 3.5) at 4°C for 1 h. After virus adsorption, cells were washed with ice-cold PBS three times, and then incubated at 37°C for 2 hr. For the control, cells were treated with 100 nM Bafilomycin A1 (Sigma Aldrich, USA) that inhibits the endosomal acidification thereby blocks the membrane fusion of the virus.

In the EI assay, A/Brisbane/10/2007 virus was pre-incubated with CT302 IgG_1,_ palivizumab or treated with Bafilomycin A1. Then the virus was used to infect A 549 cells. After virus adsorption, cells were washed with ice-cold PBS three times, and then incubated at 37°C. At 9 hr post infection, cells were fixed with 4% PFA and permeabilized with 0.25% Triton X-100. Viral nucleoprotein (NP) that is trans-located into the nucleus was stained by mouse monoclonal anti-influenza A virus nucleoprotein antibody [AA5H] (cat # ab20343, Abcam, UK). All images were acquired by Opera (PerkinElmer, MS, USA) (20x).

### Protection of mice from influenza virus infection by CT302 IgG_1_

Eight-week-old female BALB/c mice were purchased from Jackson Labs (Bar Harbor, ME, USA). Five mice of each group (described below) were anesthetized and infected via intranasal delivery with strain A/NYMC X-171 or A/Hong Kong/1/1968 (50 μl containing 1.47 x 10^2^ pfu-doses). The A/NYMC X-171 (50 μl containing 3.2 x 10^5^ EID_50_-doses) is a mouse-adapted PR8-reassortant influenza virus with surface genes from A/Brisbane/10/2007 strain [29]. Mice received 2 mg/kg or 10 mg/kg of CT302 IgG_1_ by intraperitoneal injection either 24 hr before, 24 hr after, or 48 hr after viral challenge. The antibody was diluted with 0.9% (w/v) saline solution, and PBS was used as a negative control. Mice were observed twice a day for up to 14 days, at which times the weight, clinical score, and survival were noted. Survival rates were compared among groups by using the log-rank test using GraphPad Prism (version 6.0) software. Differences were considered statistically significant when p<0.05.

### Protection of ferrets from influenza virus infection by CT302 IgG_1_

Sixteen-week-old castrated male ferrets (Mustela putorius furo) were purchased from Triple F Farms (Sayre, PA, USA) and housed in a pathogen-free environment for 7 days prior to infection with A/Brisbane/10/2007 strain. Upon receipt, ferrets were grouped during the acclimation period based on body weight in order to yield groups with approximately equal mean body weight. Before the infection, blood was collected from the ferrets and tested using the hemagglutinin inhibition (HI) assay to identify potential previous infection with influenza virus as described previously [30].

Nine ferrets of each group (see below) were infected intranasally with 1×10^6^ TCID_50_ units of A/Brisbane/10/2007 strain. TCID_50_ was defined as the tissue-culture infective dose required to cause cytopathic effects in 50% of inoculated cells. At day 1 post-infection, the infected ferrets were intravenously injected either with CT302 IgG_1_ (30 mg/kg) or an equal amount of anti-respiratory syncytial virus IgG_1_ (palivizumab) (MedImmune Inc.) as isotype-matched negative control antibody. Ferrets were monitored daily for up to 9 days post-infection. On days 1, 3, 5, and 9 after infection, 1 ml PBS containing penicillin and streptomycin was used as a nasal rinse solution to evaluate viral titers in the nasal cavity. Two ferrets from each group were randomly euthanized for lung tissue collection on days 1, 3, and 9, and three ferrets were sacrificed on day 5. Lung tissues of ferrets were mechanically homogenized in PBS containing penicillin and streptomycin using a TissueLyser (Qiagen), and homogenates were centrifuged at 13,000×*g* for 10 min to clarify supernatants. Supernatants were recentrifuged two times to assure separation from visible lung-tissue remnants. Infectious viral titers of the supernatants were determined using a TCID_50_ assay [30]. Statistical significance was assessed by paired Student's t-tests using SigmaPlot 10.0 (SPSS; Chicago, IL, USA), with p<0.05 as level of statistical significance.

### Flow cytometry

DNA fragments encoding either wild-type HA protein of A/Brisbane/10/2007 strain or A/Nanchang/933/1995-like mutants (E62K, N121T, S124G, N133D, K158E, A196V, and K276N) were chemically synthesized and ligated into pCEP4 vectors, which were transfected into human embryonic kidney (HEK293T, Invitrogen) cells as described previously [31, 32]. After 24 hr, 10 μg/ml test antibodies dissolved in flow cytometry buffer (PBS containing 1% w/v BSA and 0.05% w/v sodium azide) were incubated with transfected cells at 37°C for 1 hr. CT149 IgG_1_ was used as a positive control, and anti-respiratory syncytial virus IgG_1_ (palivizumab) (MedImmune Inc.) served as a negative control. After centrifugation at 1,000×*g* for 10 min, cells were washed twice with PBS. Cells were then incubated for 1 hr at 37°C with FITC-labeled anti-human IgG (Thermo Fisher Scientific), diluted 100-fold in flow cytometry buffer, and analyzed by flow cytometry using a FACSCanto™ II instrument (BD Biosciences; San Jose, CA, USA) equipped with a 488-nm laser. Ten thousand cells were detected per measurement with no gating, and results were analyzed using FlowJo software (TreeStar Inc.; Ashland, OR, USA).

### Visualization of HA three-dimensional surface structure

The three-dimensional structure of H3N2 HA was generated by molecular visualization system software PyMOL 1.3 (http://www.pymol.org).

## Results

### Generation of monoclonal antibodies against influenza virus H3N2

**Fig 1:**
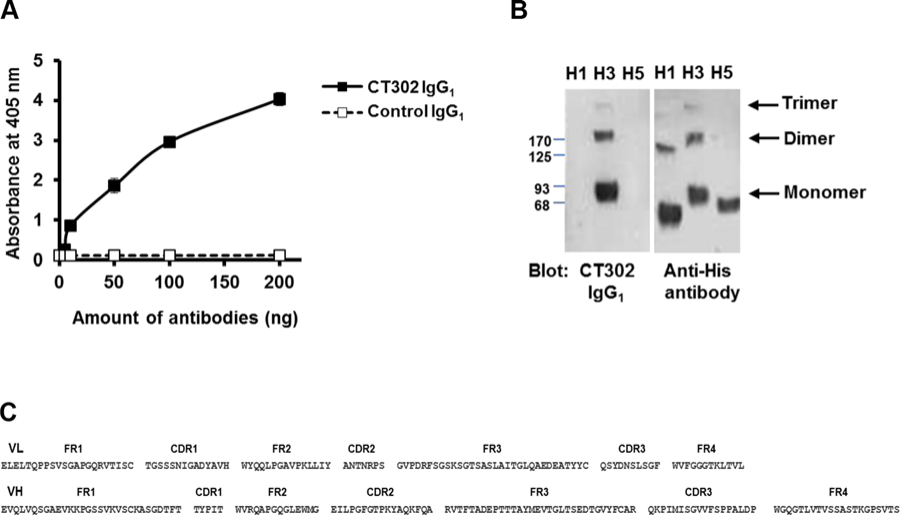
Reactivity of CT302 IgG_1_ to H3N2 trimeric HA. (A) After recombinant trimeric HA protein was coated onto 96-well plates, CT302 IgG_1_ (■) or negative-control anti-respiratory syncytial virus IgG_1_ (□) was incubated in wells as primary detection antibodies. Antibody bound to the recombinant His-tagged trimeric HA protein was detected with an HRP-conjugated anti-human IgG by addition of HRP substrate. Results represent the mean±S.D. obtained from duplicate wells per each condition. (B) Recombinant His-tagged trimeric HA proteins from H1, H3, and H5 strains were resolved by SDS-PAGE and transferred to nitrocellulose membranes, which were probed with CT302 IgG_1_ or anti-His antibody. (C) Amino acid sequences in the V_H_ and V_L_ regions of CT302 IgG_1_. HA, hemagglutinin; HRP, horseradish peroxidase.

Thirteen healthy volunteers who had not been vaccinated against influenza virus within the previous 2 years were vaccinated against the A/Uruguay/716/2007 (H3N2) strain. Almost all of the donor sera showed increased antibody titers after vaccination (data not shown). Using mRNA from mononuclear cells of the vaccinated group, we generated a scFv library with a complexity of 1.65×10^10^ clones. Next, we performed four rounds of biopanning and enzyme immunoassay to select phage bound to recombinant trimeric HA protein of the A/Brisbane/10/2007 strain, which shares the same amino acid sequence (except A138) with A/Uruguay/716/2007 (accession number CY121632.1). Twenty positive clones were identified among 192 colonies initially tested, and sequence analysis revealed 14 positive clones with unique amino-acid sequences. From these 14 positive clones, we selected the five most reactive clones to generate human anti-HA IgG_1_ antibodies. Reactivity of the antibodies to HA was confirmed by enzyme immunoassay and immunoblot analysis (Fig 1A and 1B, respectively).

The microneutralization assay was used to test the neutralizing activity of these five anti-HA IgGs against H3N2 influenza virus. We tested three H3N2 influenza viruses: A/Brisbane/10/2007, A/Hong Kong/1968, and A/Puerto Rico/8/1934 strains. All five antibody clones efficiently neutralized only the A/Brisbane/10/2007 strain, and we selected the three antibodies with the greatest neutralization activity. Among these three antibody clones, we selected CT302 IgG_1_ for further studies because it expressed the highest yield. The amino-acid sequences of the V_H_ and V_L_ variable regions of CT302 IgG_1_ are shown in Fig 1C. In real-time interaction analysis of CT302 IgG_1_, using HA-coated chips (recombinant His-tagged trimeric HA from A/Brisbane/10/2007 and A/Wisconsin/67/2005 strains), we determined that the *K*_*D*_ constants were 6.06±0.03×10^−10^ and 8.55±0.74×10^−9^, respectively (Table 1).

**Table 1:** Affinity constants (*K*_*a*_, *K*_*d*_) of CT302 IgG_1_ determined by real-time interaction analysis.

Immobilized HA protein	*K*_*D*_ (M)	*K*_*a*_ (M^-1^S^-1^)	*K*_*d*_ (S^-1^)
A/Brisbane/10/2007	6.06±0.03×10^−10^	2.93±0.03×10^5^	1.78±0.01×10^−4^
A/Wisconsin/67/2005	8.55±0.74×10^−9^	2.91±0.07×10^5^	2.45±0.15×10^−3^

*K*_*a*_, association rate constant; *K*_*d*_, dissociation rate constant; *K*_*D*_, dissociation equilibrium constant calculated based on *K*_*d*_/*K*_*a*_; M, molarity.

### *In vitro* neutralizing and blocking activity of CT302 IgG_1_ against diverse influenza A strains

To determine the neutralizing activity of CT302 IgG_1_ against a spectrum of seasonal H3N2 viruses circulating during the past decade, we performed additional microneutralization assays with nine different H3N2 viruses isolated between 1968 and 2007. CT302 IgG_1_ neutralized several recently identified H3N2 subtypes of influenza A viruses: A/Brisbane/10/2007, A/Wyoming/3/2003.rg, A/Panama/2007/1999, and A/Sydney/5/1997 strains (Table 2). Interestingly, CT302 IgG_1_ did not neutralize H3N2 strains identified during or before 1995, such as A/Nanchang/933/95, A/Johannesburg/33/1994-R, A/Beijing/32/1992-R, A/Beijing/353/1989-X109, or A/Hong Kong/1968 strains.

**Table 2:** Microneutralization activity of CT302 IgG_1_ against influenza virus strains.

Strain	MN titer (μg/ml)^a^
A/Brisbane/10/2007	0.009
Wyoming/3/2003.rg	0.0024
A/Panama/2007/1999	0.039
A/Sydney/5/1997	0.019
A/Nanchang/933/1995	>20^b^
A/Johannesburg/33/1994 R	>20
A/Beijing/32/1992-R	>20
A/Beijing/353/1989-X109	>20
A/Hong Kong/1/1968	>20

^a^MN (microneutralization) titers are expressed as the lowest concentration of purified CT302 IgG_1_ that completely neutralized the influenza virus.

^b^No detectable neutralization activity at the concentration of 20 μg/ml.

**Fig 2:**
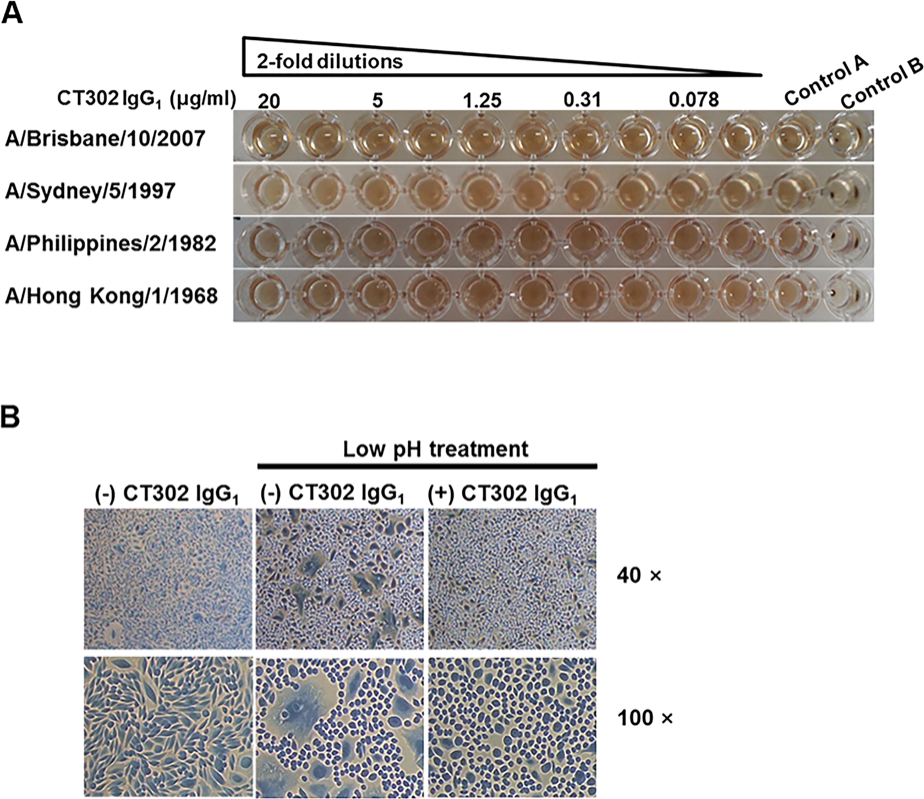
Inhibition of hemagglutination and cell fusion by CT302 IgG_1_. (A) Two-fold dilutions of CT302 IgG_1_ (over the range of 20 to 0.039 μg/ml in PBS) were added to wells harboring 8 hemagglutination units of influenza virus, and chicken red blood cells (RBCs) were added to a final concentration of 0.5%. Control A wells contained only RBCs and influenza virus, without CT302 IgG_1_. Control B wells contained only RBCs, without influenza virus or CT302 IgG_1_. (B) CHO cells expressing HA protein of A/Brisbane/10/2007 strain were incubated with CT302 IgG_1_ and then exposed to low-pH medium (pH 5.0) to induce membrane fusion. As controls, cells not treated with CT302 IgG_1_ but exposed to low pH, or cells not treated with CT302 IgG_1_ and not exposed to low pH, are shown. Magnification: 40×, 100×. TCID_50_, tissue-culture infective dose required to cause cytopathic effects on 50% of inoculated cells.

Next, we performed a hemagglutination inhibition assay to assess potential CT302 IgG_1_ inhibition of viral attachment to the sialic-acid receptor on the surface of target cells, and we performed a membrane-fusion assay to investigate CT302 IgG_1_’s potential inhibition of influenza virus HA-induced target-cell membrane fusion. The hemagglutination inhibition assay utilized chicken RBCs to test CT302 IgG_1_ against four influenza virus strains: A/Brisbane/10/2007, A/Sydney/5/1997, A/Philippines/2/1982, and A/Hong Kong/1968 strains. Even at the highest concentration tested (20 μg/ml), CT302 IgG_1_ did not show any hemagglutination-inhibitory activity against any of the influenza strains tested (Fig 2A), while a control antibody effectively inhibited hemagglutination in a parallel experiment (data not shown). In the absence of the influenza virus, CT302 IgG_1_ did not induce the hemagglutination of chicken or turkey RBCs (data not shown).

**Fig 3:**
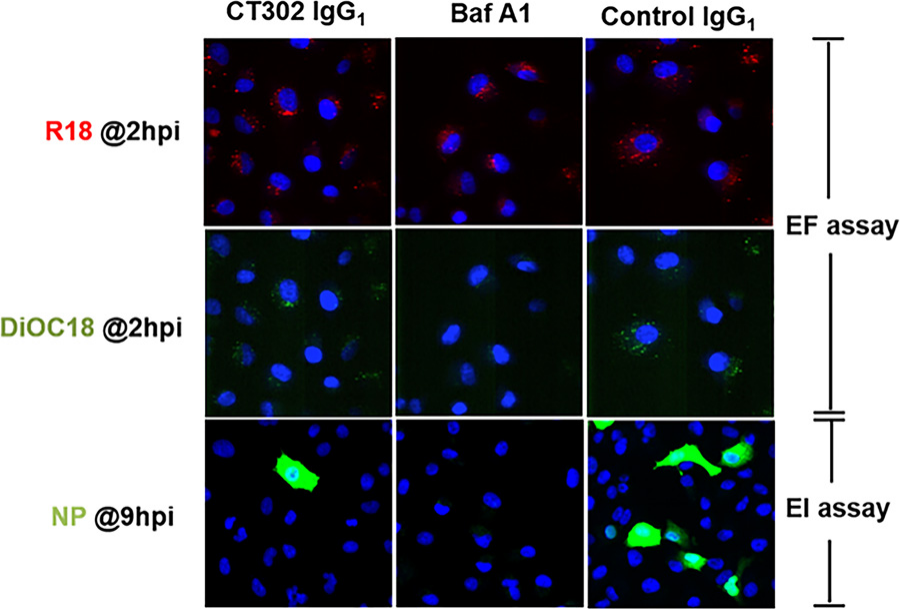
Effect of CT302 IgG_1_ on the membrane fusion and the traffic of viral nucleoprotein to nucleus. Entry fusion (EF) assay: Virus particles were labeled with DiOC18 and R18, and were allowed to enter CT302 IgG1-, Bafilomycin A1- or control IgG1-treated cells. Fusion of viral and vacuolar membranes of cells triggered dequenching of DiOC18 (green) signal co-localized with the R18 (Red) signal. Nuclear import (EI assay): In the CT302 IgG_1_-, BafilomycinA1- or control IgG_1_-treated cells, virus particles were allowed to enter nucleus. Incoming NP proteins (green) were detected within the nucleus (blue) by the anti-NP antibody. Magnification: 20×.

In the cell-membrane fusion assay using CHO cells that overexpressed HA protein from A/Brisbane/10/2007 strain, CT302 IgG_1_ at a concentration of 10 μg/ml completely inhibited HA-dependent CHO-cell membrane fusion (Fig 2B). In viral entry fusion (EF) assay, CT302 IgG_1_ at the concentration of 12.5 mg/ml did not block fusion of viral and vacuolar membranes of cells (Fig 3), which is contradictory to the result of the cell-based membrane fusion assay in this study.

To monitor the traffic of viral NP to nucleus, we followed the nuclear accumulation of NP using NP monoclonal antibody in the EI assay. In this assay we found the nuclear accumulation of viral NP in the cells treated with CT302 IgG_1_ was lower than that of NP in the cells treated with the control IgG1 (Fig 3).

### *In vivo* neutralizing activity of CT302 IgG_1_ on influenza infectivity

**Fig 4:**
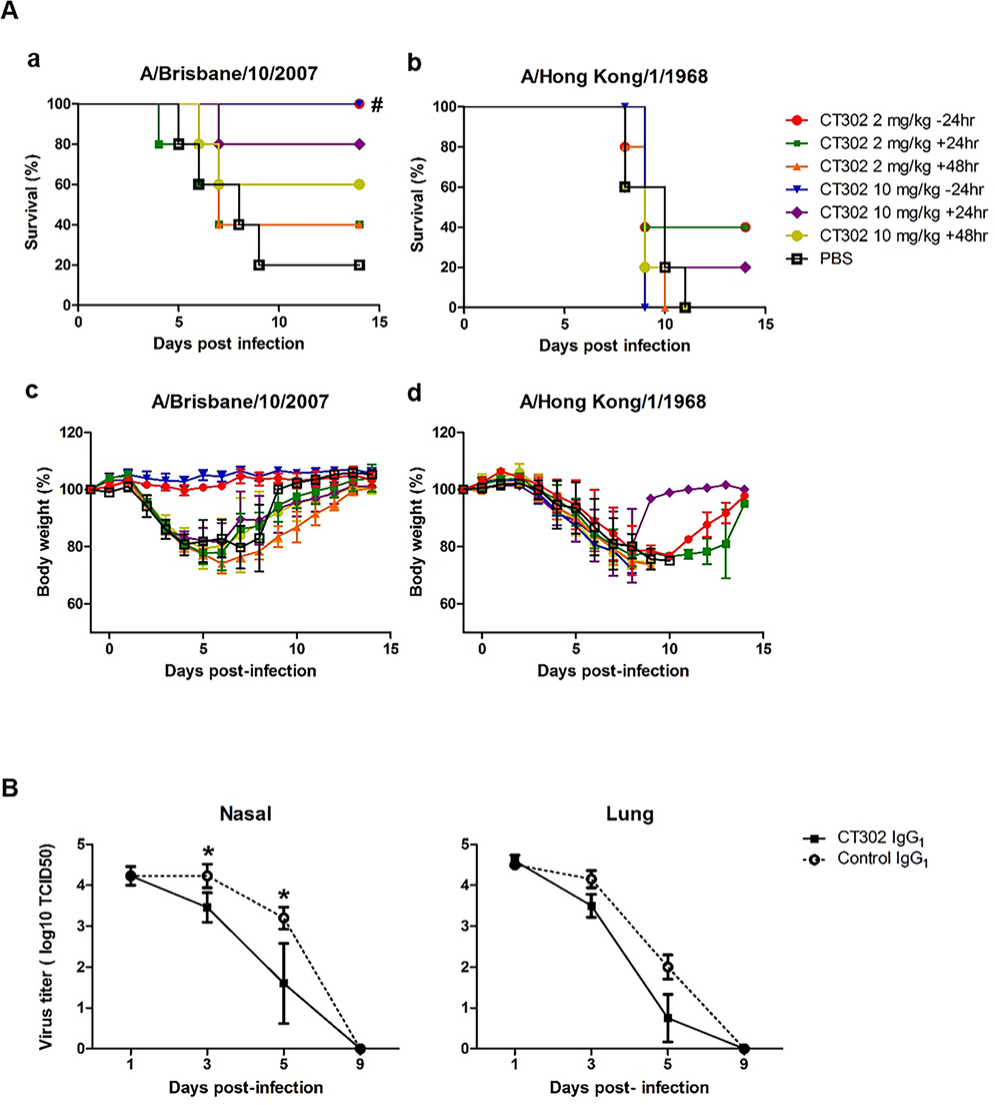
Protective and therapeutic effects of CT302 IgG_1_ in influenza virus-infected mice and ferrets. (A) CT302 IgG_1_ was administered intraperitoneally to mice at -24, +24, or +48 hr from infection with A/NYMC X-171 strain (mouse-adapted A/Brisbane/10/2007 strain) (a, c) or A/Hong kong/1/1968 (b, d), and survival rate (a, b) and body weight change (c, d) were monitored twice daily up to 14 days. (B) Ferrets were challenged with the influenza virus and treated intravenously either with 30 mg/kg CT302 IgG_1_ or control IgG_1_ after 1 day. Subsequently, viral titers in the nasal fluid and lung tissue were determined. ^#^p<0.05 (vs. PBS) determined by log-rank (Mantel-Cox) test. *p<0.05 (vs. control) on day 5, determined by Student’s t-test. TCID_50_, tissue-culture infective dose required to cause cytopathic effects on 50% of inoculated cells.

The preventive potential of CT302 IgG_1_ against influenza virus infection was tested in mice using the A/NYMC X-171 strain (mouse-adapted A/Brisbane/10/2007 strain) and A/Hong Kong/1/1968. Influenza infection in PBS-treated control mice resulted in only 20% survival after 9 days, which was consistent throughout the 14-day experiment. All mice pretreated before influenza virus infection with CT302 IgG_1_, at either 2 or 10 mg/kg, survived through the two-week observation period. This demonstrates that CT302 IgG_1_ has a potent preventative effect against infection by strain A/NYMC X-171 (p<0.05; hazard ratio, 0.08; 95% confidence interval, 0.01–0.59). To explore the therapeutic potential of CT302 IgG_1_, mice were first inoculated with influenza virus and then treated with CT302 IgG_1_. After administering 10 mg/kg CT302 IgG_1_ at 24 or 48 hr postinfection, mouse survival rates were 80% (p = 0.08) or 60% (p = 0.28), respectively, at 14 days postinfection. When mice were treated either 24 or 48 hr postinfection with 2 mg/kg CT302 IgG_1_, the survival rates were 40% (p = 0.08 and 0.06, respectively) (Fig 4A. a). In correlation with microneutralization result, CT302 IgG_1_ did not show any prophylactic or therapeutic efficacy in mice infected with A/Hong Kong/1/1968 (Fig 4A.b).

To evaluate the efficacy of CT302 IgG_1_ in suppressing viral replication, ferrets were first infected with A/Brisbane/10/2007 strain and then treated intravenously on day 1 postinfection with either CT302 IgG_1_ or control anti-respiratory syncytial virus IgG_1_ (30 mg/kg) (Fig 4B). The nasal-cavity viral titers of infected ferrets treated with CT302 IgG_1_ were significantly lower on days 3 and 5 postinfection than in ferrets treated with control anti-respiratory syncytial virus IgG_1_ (p<0.05 at both time points). The viral titers in lung tissue were also significantly lower at day 5 postinfection in CT302 IgG_1_-treated ferrets than in animals treated with control anti-respiratory syncytial virus IgG_1_ (p<0.05).

### Epitope mapping of CT302 IgG_1_

**Fig 5:**
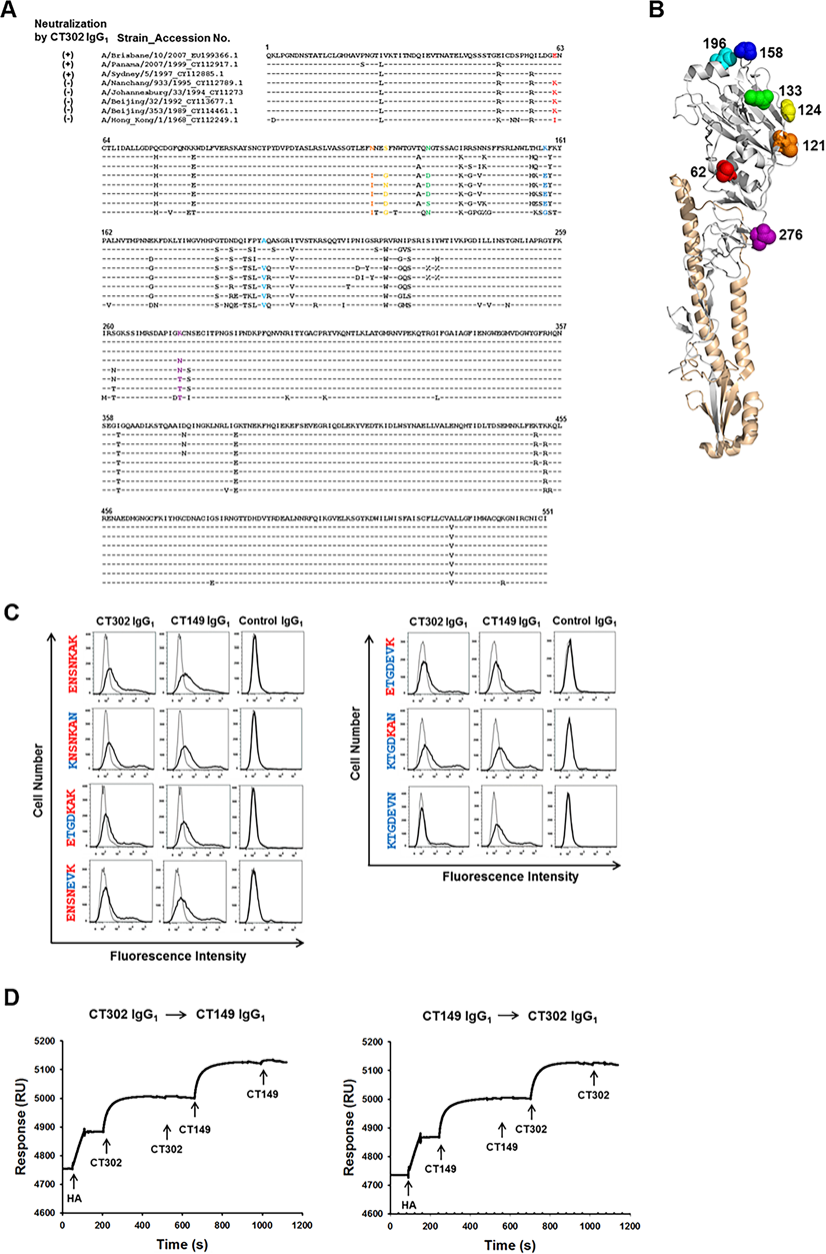
Binding activity of CT302 IgG_1_ to HA mutants. (A) Globular head domain of HA protein is composed of residues 52 to 277. The amino-acid sequences for HA protein were downloaded from the Influenza Virus Resource at the National Center for Biotechnology Information (NCBI). The NCBI accession numbers are indicated next to each strain name. (B) Locations of the seven residues (62, 121, 124, 133, 158, 196, and 276) that vary among the influenza strains that are either neutralized or not neutralized by CT302 IgG_1_ are indicated in the three-dimensional structure of HA. The illustrations were constructed on H3N2 HA (Protein Data Bank accession code 4FNK) using molecular-visualization system software PyMOL 1.3. (C) HEK293T cells expressing wild-type or mutant HA were analyzed by flow cytometry using CT302 IgG_1_, CT149 IgG_1_ (an anti-stem-region IgG_1_), or an anti-respiratory syncytial virus IgG_1_ (negative control). (D) In real-time interaction analysis, sensor chips were first conjugated with anti-His antibody. Next, His-tagged recombinant HA protein of A/Brisbane/10/2007 stain was loaded, and the interaction of HA protein with CT302 IgG_1_ or CT149 IgG_1_ was examined by sequentially applying these antibodies. Either antibody bound after presaturation with the other antibody, indicating that these two antibodies bind noncompetitively to different epitopes. RU, resonance units.

Amino-acid residues that differed between viral strains A/Sydney/5/1997 (neutralized by CT302 IgG_1_) and A/Nanchang/933/1995 (not neutralized by CT302 IgG_1_) were found at positions 62 (E or K), 121 (N or T), 124 (S or G), 133 (N or D), 158 (K or E), 196 (A or V), and 276 (K or N) (Fig 5A and 5B).

To determine the effect of amino acid substitutions on the binding of CT302 IgG_1_ to HA of the A/Brisbane/10/2007 strain, we expressed six HA mutants on the cell surface of HEK293T cells. Expression levels of HA mutants were confirmed by flow cytometry using CT149 IgG_1_, which targets the HA stem region, and using an isotype-matched human IgG_1_ antibody as negative-control (Fig 5C). CT302 IgG_1_ strongly reacted to HA of A/Brisbane/10/2007 strain (ENSNKAK) but did not react to the KTGDEVN mutant, whose sequence is exactly homologous to the HA of the A/Nanchang/933/1995 strain. The CT302 IgG_1_ showed notably decreased reactivity to ENSNEVK, ETGDEVK, and ETGDKAK mutants, while minimal or no decreased reactivity was observed using KNSNKAN and KTGDKAN mutants (Fig 5C). These data suggest that the 121N, 124S, 133N, 158K, and 196A residues are involved in the interaction between HA and CT302 IgG_1_.

### Real-time interaction analysis

Because all differing residues between the two influenza strains that showed strong (A/Sydney/5/1997 strain) or no reactivity (A/Nanchang/933/1995 strain) to CT302 IgG_1_ were located in the HA globular head domain, we assumed the binding site of CT302 IgG_1_ was localized in the globular head domain. To confirm this, we determined if CT302 IgG_1_ could bind to HA in the presence of another neutralizing antibody, CT149 IgG_1_, which binds to the HA stem region. Only two epitopes on the HA stem region related to neutralizing-antibody binding have been identified, and these epitopes share some overlapping residues [33]. Therefore, if CT302 IgG_1_ does not compete with CT149 IgG_1_ for HA binding, we would conclude CT302 IgG_1_ binds specifically to the HA globular head domain.

In real-time interaction analysis, recombinant His-tagged HA protein was conjugated to the sensor chip surface. After conjugation, a sufficient amount of CT302 IgG_1_ was injected to completely cover the chip-bound recombinant His-tagged HA protein, which we confirmed by observing no additional increase in resonance after another injection of CT302 IgG_1_ (Fig 5D, left graph). After establishing maximal CT302 IgG_1_ binding, we injected CT149 IgG_1_, which successfully bound to recombinant His-tagged HA protein, indicating that these two antibodies noncompetitively bind to different HA epitopes. In a parallel experiment, the sequence of injecting these two antibodies was reversed. CT302 IgG_1_ successfully bound to chip-immobilized recombinant His-tagged HA that was saturated with CT149 IgG_1_ (Fig 5D, right graph), also indicating noncompetitive HA binding by these two antibodies.

## Discussion

Four main antiviral mechanisms are known to inhibit influenza infection by monoclonal antibodies that target HA [34]. First, antibodies such as S139/1, CH65, 1F1, and HC63 disrupt virus attachment to sialic acid residues on the surface of the host cell, thereby blocking viral entry [35–37]. These antibodies are generally found to inhibit hemagglutination. Second, anti-HA globular head-domain monoclonal antibodies, such as CR8071 and CR8033, inhibit progeny virion released from infected cells [38], and different antibody-binding epitopes exist among diverse influenza strains. For example, CR8033 has hemagglutination-inhibitory activity, blocking target-cell infection by influenza virus when preincubated with the B/Florida/4/2006 strain, but has no effect on strain B/Malaysia/2506/2004 infectivity. CR8071 does not prevent cell infection by either influenza virus [38]. Third, some anti-HA stem-region monoclonal antibodies, such as C179, CR6261, F10, and CR8020, interfere with viral membrane fusion to target cells in cell-based membrane fusion assay [35] without interfering with receptor interactions that mediate virion attachment. Antibodies CR6261 and F10 inhibit HA conformational changes, while CR8020 inhibits cleavage by trypsin [16]; neither of these antibodies inhibits hemagglutination. Fourth, several anti-stem antibodies, such as 6F12, F16, 2G02, 2B06, and 1F02, require FcγR interactions to protect the host cell from influenza virus [34]. These antibodies depend on Fc interactions with FcγR to achieve antiviral activity and can mediate antibody-dependent cytotoxicity of infected cells.

The anti-HA globular head-domain antibody CT302 IgG_1_, explored in our current study, did not block the membrane fusion between the virus envelope and endosomal membrane as shown in our EF assay, while it blocked the membrane fusion in cells displaying HA protein in the cell fusion assay. As the EF assay represent physiologically more relevant condition of influenza virus infection process, we believe that CT302 IgG_1_ does not block the membrane fusion process.

On the other hand, the CT302 IgG_1_ inhibited nuclear import of NP in EI assay. Currently we do not know the mechanism by which CT302 IgG_1_ can block the nuclear import of NP. Tripartite motif (TRIM)-containing proteins, especially TRIM5α, act to repress viral replication. TRIM5α has two known functions in the cell. It has a role as a defensive retroviral capsid-binding protein that can inhibit retroviral infection. The second role is as an E3 ubiquitin ligase that promotes NF-kB and AP-1 transcription factor signaling [39]. The general accepted model of TRIM5α restriction is that when bound to capsids, it recruits proteasomes that potentiate disassembly of the capsid. However, in the absence of the second stage of restriction that is proteasome-dependent, the fate of the non-infectious TRIM5α–capsid complex is uncertain. Although the viral uncoating is not rapid and the virus has sufficient time and resources to undergo reverse transcription, it is prevented from entering the nucleus and forming DNA circles [39, 40]. Similarly, CT302IgG_1_ prevents NP to nucleus. Although the mechanisms by which CT302 IgG_1_ and TRIM5α block nuclear import is not yet clear, it is clear that there is a mechanism that inhibit the viral infection process between membrane-fusion and nuclear importation.

## Supporting Information

S1 ARRIVE Checklist (DOCX)
