# Supplementary material for: An Anti-Influenza Virus Antibody Inhibits Viral Infection by Reducing Nucleus Entry of Influenza Nucleoprotein
Source: PLoS One. 2015 Oct 29;10(10):e0141312. doi: 10.1371/journal.pone.0141312 (PMC4626144; doi:10.1371/journal.pone.0141312)

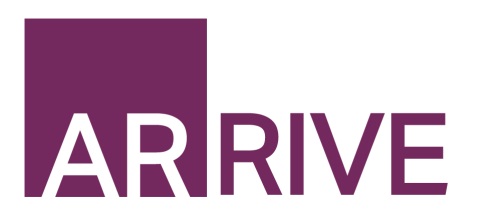


The ARRIVE Guidelines Checklist

Animal Research: Reporting In Vivo Experiments

Carol Kilkenny^1^, William J Browne^2^, Innes C Cuthill^3^, Michael Emerson^4^ and Douglas G Altman^5^

*^1^The National Centre for the Replacement, Refinement and Reduction of Animals in Research, London, UK, ^2^School of Veterinary Science, University of Bristol, Bristol, UK, ^3^School of Biological Sciences, University of Bristol, Bristol, UK, ^4^National Heart and Lung Institute, Imperial College London, UK, ^5^Centre for Statistics in Medicine, University of Oxford, Oxford, UK.*

|  | | ITEM | RECOMMENDATION | Section/ Paragraph |
| --- | --- | --- | --- | --- |
| 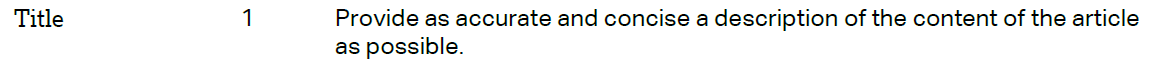 | | | N/A |  |
| 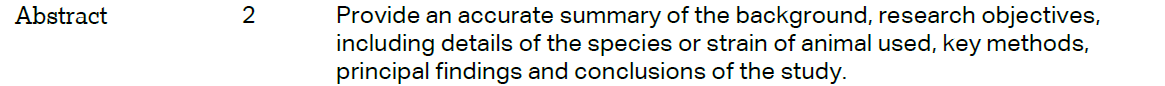 | | | N/A |  |
| INTRODUCTION | | |  |  |
| 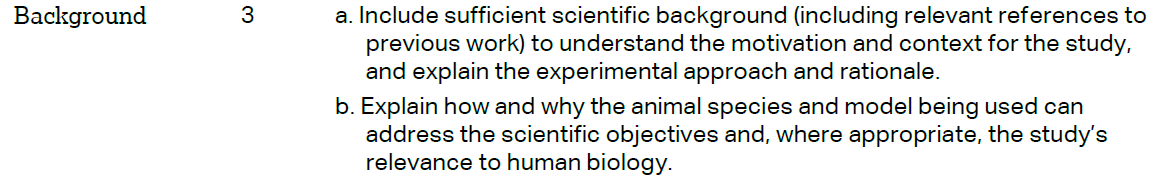 | | | N/A |  |
| 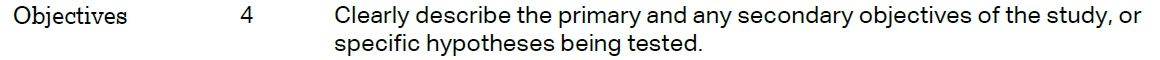 | | | Introduction Paragraph 4 |  |
| METHODS | | |  |  |
| 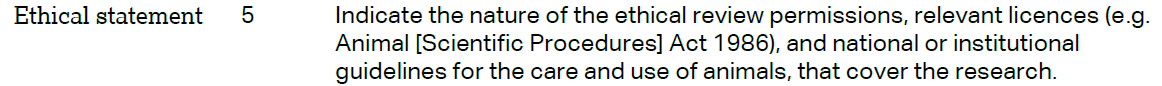 | | | Materials and Methods / Paragraph 2 and 3 (Ethics Statement) |  |
| 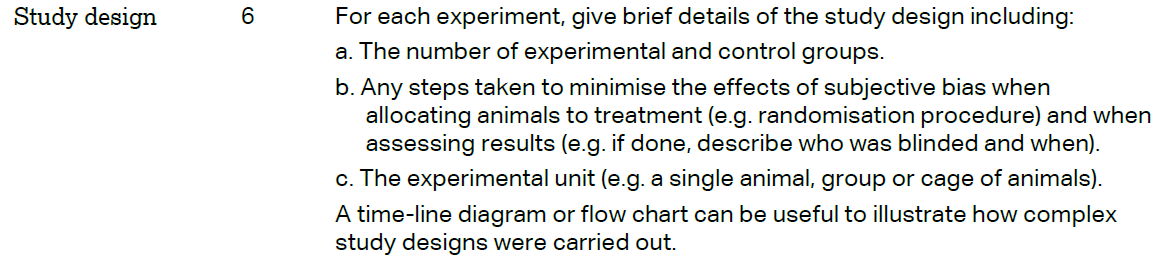 | | | Materials and Methods/ Paragraph 13 thorough 15 (Applicable only to ‘a’ & ‘c’) |  |
| 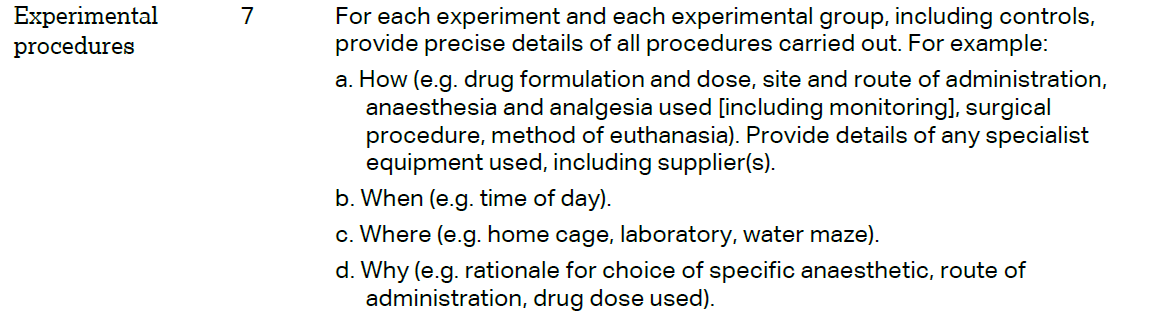 | | | Materials and Methods / Paragraph 13 thorough 15 (Applicable to ‘a’, ‘b’ and partially ‘c’) |  |
| 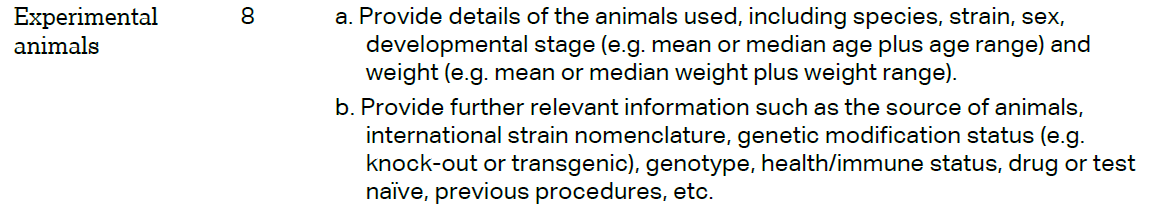 | | | Materials and Methods / Paragraph 13 thorough 15 (applicable to ‘a’) |  |

| 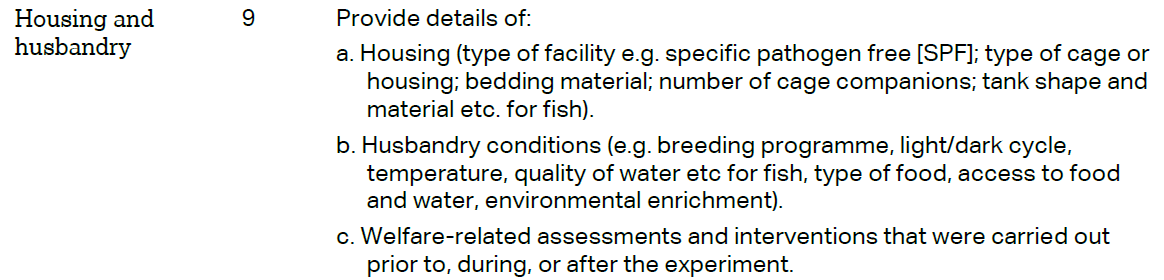 | Materials and Methods / Paragraph 2 and 3 (Ethics Statement, applicable to ‘c’) | |
| --- | --- | --- |
| 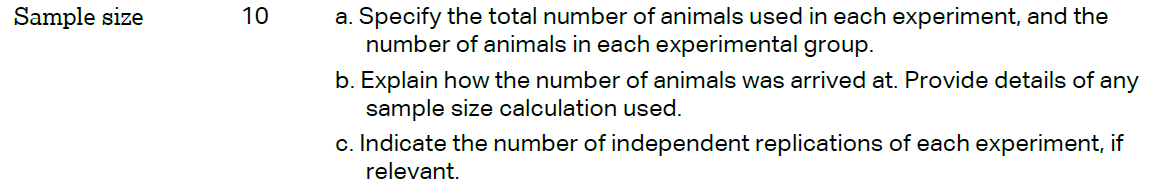 | Materials and Methods/ Paragraph 13 thorough 15 (applicable to ‘a’) | |
| 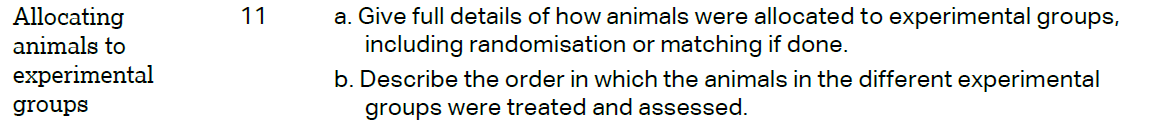 | Materials and Methods/ Paragraph 13 thorough 15 (applicable to ‘a’) | |
| 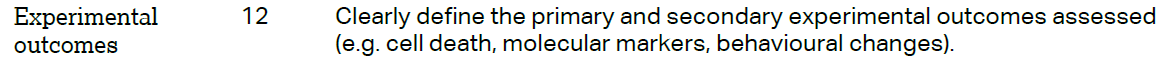 | Materials and Methods/ Paragraph 13 thorough 15 | |
| 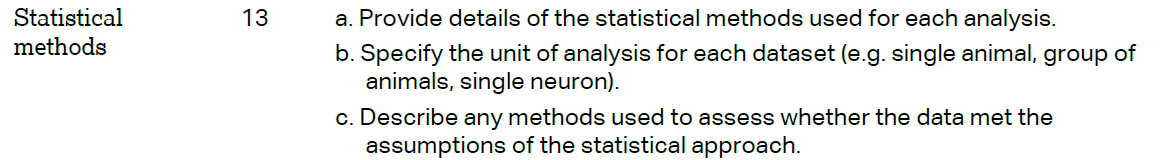 | Materials and Methods/ Paragraph 13 thorough 15 (applicable to ‘a’) | |
| RESULTS |  | |
| 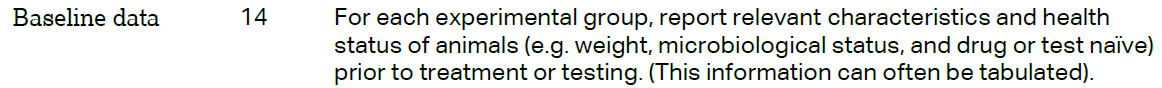 | Figure3A (c and d) | |
| 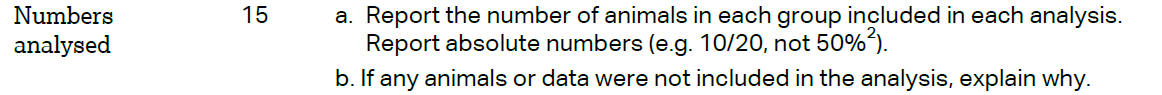 | N/A | |
| 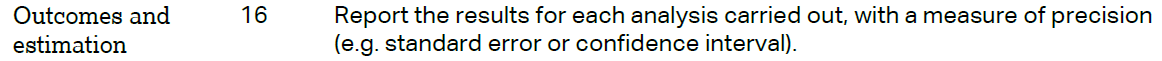 | Results/ Paragraph 5 and 6 | |
| 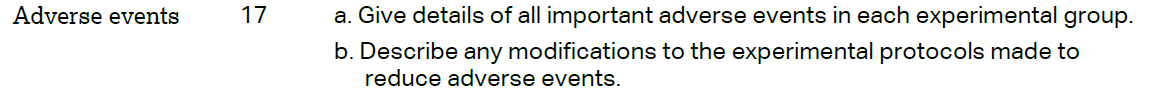 | N/A | |
| DISCUSSION |  | |
| 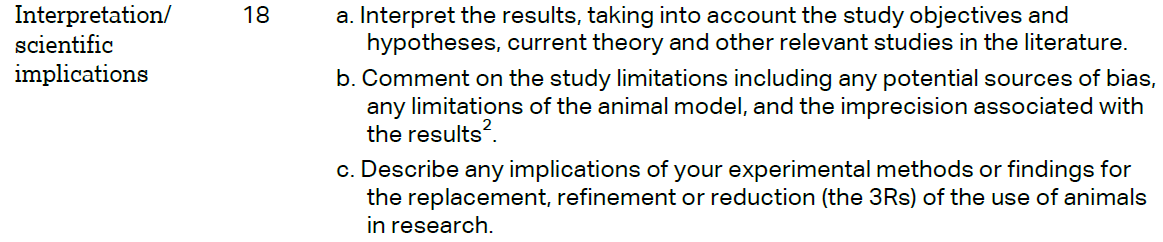 | N/A | |
| 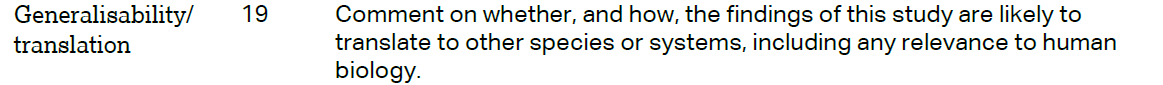 | N/A | |
| 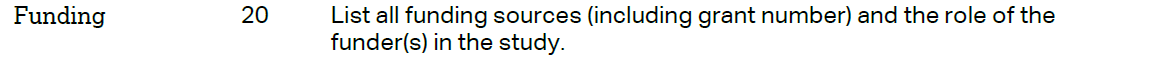 | | N/A |


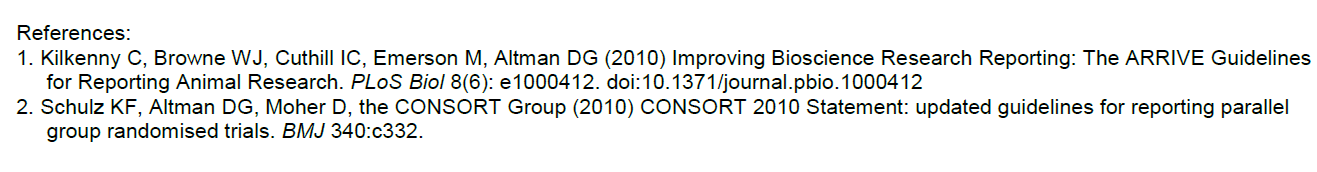

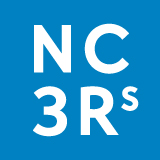

Supplement: S1 ARRIVE Checklist — (DOCX) [file pone.0141312.s001.docx]
